# Cross-Domain Object Detection by Dual Adaptive Branch

**DOI:** 10.3390/s23031199

**Published:** 2023-01-20

**Authors:** Xinyi Liu, Baofeng Zhang, Na Liu

**Affiliations:** 1 The School of Computer Science and Engineering, Tianjin University of Technology, No. 391 Bin Shui Xi Dao Road, Tianjin 300384, China; 2 Tianjin Key Laboratory for Control Theory and Applications in Complicated System, Tianjin University of Technology, No. 391 Bin Shui Xi Dao Road, Tianjin 300384, China

**Keywords:** cross-domain, object detection, domain adaptive, dual-adaptive

## Abstract

The object detection task usually assumes that the training and test samples obey the same distribution, and this assumption is not valid in reality, therefore the study of cross-domain object detection is proposed. Compared with image classification, the cross-domain object detection task presents the greater challenge, which requires both accurate classification and localization of samples in the target domain. The teacher–student framework (the student model is supervised by pseudo-labels from the teacher model) has produced a large accuracy improvement in cross-domain object detection. Feature-level adversarial training is used in the student model, which allows features in the source and target domains to share a similar distribution. However, the direction and gradient of the weights can be divided into domain-specific and domain-invariant features, and the purpose of domain adaptive is to focus on the domain-invariant features while eliminating interference from the domain-specific features. Inspired by this, we propose a teacher–student framework named dual adaptive branch (DAB), which uses domain adversarial learning to address the domain distribution. Specifically, we ensure that the student model aligns domain-invariant features and suppresses domain-specific features in this process. We further validate our method based on multiple domains. The experimental results demonstrate that our proposed method significantly improves the performance of cross-domain object detection and achieves the competitive experimental results on common benchmarks.

## 1. Introduction

With the development of deep neural networks, many computer vision tasks have achieved great success. The convolutional neural network-based object detection has achieved excellent performance on various benchmark datasets. However, these successes also depend on a large amount of labeled data [1]. The collection of this data is very costly and time-consuming. Moreover, the performance of object detection models trained based on annotated data might be substantially degraded in a real scenario, and the main reason is a change in the captured view, appearance, background, lighting, or image quality. For these changing visual conditions, some work has begun to investigate the adaptive method [2]. Domain adaptive methods [3] can transfer knowledge from labeled source domains to unlabeled target domains, and it is a more cost-effective and functional option than annotating enough object samples. Therefore, the domain adaptive methods have a widely application in many fields.

The common domain adaptive methods include global feature adaptation [4], instance feature adaptation [5], and local feature adaptation [6]. The global feature adaptation easily confuses the object features in different categories, since each image contains multiple objects. Instance feature adaptation easily confuses the foreground and background features, since the detector output on the target domain is unstable, and most of the predicted foreground is actually background. Local feature adaptation is typically used to address the distribution shifts on the lower semantic levels. Domain adaptive in object classification only requires alignment to features in the same category of objects. However, which part of the features to align has also become a critical study problem in the object detection task because there is the uncertainty about the location of object.

**Figure 1:**
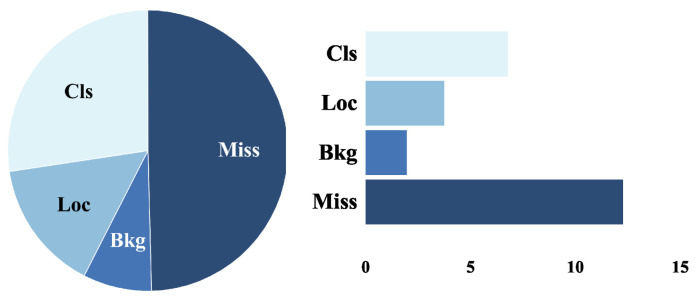
The main error sources in cross-domain object detection.

In cross-domain object detection task, it generally contains a domain-invariant feature and a domain-specific feature. The domain-invariant feature is expressed as the composition of the object, and the domain-specific feature is expressed as whether the object has clear boundaries. As shown in Figure 1, we have analyzed the meaningful error types in cross-domain object detection. These error types are related to overall performance to minimize any confounding variables. This allows one to improve the interpretability of design decisions and to describe more clearly the strengths and weaknesses of the model. The pie chart shows the relative contribution of each error, while the bar plots show their absolute contribution. We can observe that the prediction results usually contain a large number of errors and false positives. This method cannot focus on domain-invariant feature alignment. If features are aligned as a whole instead of distinguishing between domain-invariant and domain-specific features, it will be detrimental to solve the errors and false positives problem in cross-domain object detection.

A desirable domain adaptive method should achieve the alignment of the domain-invariant features. Otherwise, it might have a “negative migration” effect on the target domain. The distribution difference metric function can minimize the difference between source and target domains. We can assume that the domains have the same or similar feature distributions when the difference is sufficiently minimal. However, considering the feature alignment method in this way, we find that it does not distinguish the domain-invariant and domain-specific features of the downstream task. Inspired by the above, we propose to suppress domain-specific features in the model. There is more focus on learning domain-invariant features on higher-level semantic space.

In this work, we propose a novel cross-domain object detection network with DAB structure. The purpose is to train a well performing model on a target domain with unknown domain annotations. We use adversarial learning and mutual learning to improve the detection performance of the target domain. Our model consists of two modules: a target-domain teacher model and a cross-domain student model. In the student model, we propose a domain-invariant feature alignment branch and a domain-specific feature suppression branch. A distribution difference measure function that focuses on domain invariant feature alignment while suppressing domain specific features is proposed. We map the features to a high-dimensional space and use discriminators with gradients for adversarial learning which adjusts the distribution differences between the source and target domains in the student model.

The main contributions of this paper are as follows:

(1) A novel cross-domain object detection framework with dual adaptive branch is designed, which we called DAB. This framework utilizes two branches, domain-invariant feature alignment branch and domain-specific feature suppression branch, to overcome errors and false positives in cross-domain object detection.
(2) The feature alignment branch and feature suppression branch are designed, respectively. With the purpose of domain-invariant feature alignment, we propose to map the features into a high-dimensional space and restrict the gradient using a distribution difference measure function, which minimizes the difference of domain-invariant features between two domains. With the purpose of domain-specific feature suppression, we propose to impose constraints on domain-specific features, which eliminate the influence of domain-specific features on cross-domain object detection.
(3) The extensive experiments are conducted on various cross-domain benchmarks, and the experimental results demonstrate that our method achieves a significant performance improvement.

## 2. Related Works

Object Detection. Early object detection methods were based on the sliding-window methods, which apply the hand-crafted features and classifiers on dense image grids to find objects [7,8]. However, the traditional hand-crafted feature extraction method for object detection has some limitations, such as poor robustness to changing the objects, high time complexity, and redundant detection window. With the arrival of the deep convolutional neural network, one can solve the problems of traditional hand-crafted feature extraction methods and improve the detection speed and accuracy of object detection. The object detection task is quickly dominated by convolutional neural network (CNN), which can be divided into the two-stage object detection [9,10,11] and one-stage object detection [12,13,14,15,16].

Cross domain Object Detection. The purpose of cross-domain object detection is to detect objects in different domains. Xu et al. [16] have proposed to alleviate the domain shift problem of deformed component-based models (DPMs) by introducing adaptive support vector machine (SVM). Raj et al. [17] have proposed the subspace alignment methods to align the features extracted by using R-CNN models. The works mentioned above are either not trained in the end-to-end manner or focused on a specific case. Cross-domain object detection methods can be generally divided into two categories, namely, adversarial feature alignment [4,5,6,18,19,20,21,22,23,24,25,26] and self-training [27,28,29,30,31]. Besides the standard cross-domain object detection, the tasks of passive and multi-source have been studied in [21,32,33], respectively. In addition, refs. [34,35] have explored the problem of domain generalizability for object detection. This direction of research was first carried out by Chen et al. [5], who proposed a domain-adaptive faster R-CNN, which reduces the difference between image-level and instance-level distributions by embedding adversarial feature adaptation in a two-stage detection pipeline. Saito et al. [6] have proposed to align shallow local perceptual fields with deeper image-level features, i.e., strong local alignment and weak global alignment. Additionally, He et al. [35] have proposed a hierarchical domain feature alignment module and a weighted GRL to reweight the training samples. Kim et al. [36] have randomly expanded the source and target domains into multiple domains and solved the adaptation problem from the perspective of domain diversification. It addresses the issue of adaptability from the perspective of domain diversity. Ref. [37] has proposed a novel approach to domain adaption for object detection to mine the discriminative regions and focus on aligning them across both domains. Ref. [38] have adopted multi-level domain feature alignment. Ref. [39] has utilized classification consistency of image-level and instance-level predictions with the assistance of a multi-label classification model. Ref. [20] has proposed a center-aware feature alignment method that enables the discriminator focus on features from object regions. Refs. [4,25] have emphasized the different strategies for dealing with foreground and background features. Another popular methodology [27,28,39,40,41] is dedicated to address the problem of inaccurate labels in the target domain.

In summary, these methods have not properly addressed the potential contradiction between shift-ability and discrimin-ability in cross-domain object detectors. Therefore, we propose to design a novel dual adaptive branch, which addresses the domain-invariant feature alignment and domain-specific feature suppression in the cross-domain object detection task.

## 3. Proposed Method

### 3.1. Framework Overview

**Figure 2:**
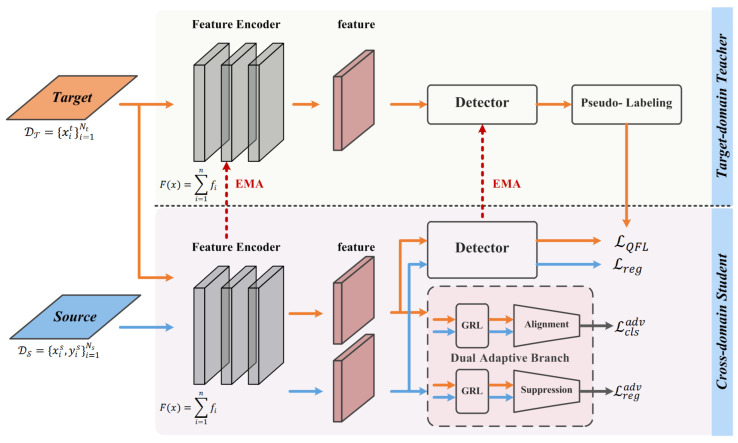
Framework Overview. This framework consists of two parts: a target-domain teacher model and a cross-domain student model. In target-domain teacher model, the target domain images are feeding into the teacher model to generate pseudo-labels. In cross-domain student model, we propose a DAB structure. With the purpose of feature alignment branch, the features are mappes into a high-dimensional space, and they restrict the gradient using a distribution difference measure function, which minimizes the difference of domain-invariant features between two domains. With the purpose of feature suppression branch, the domain-specific features are constrainted, which eliminate the influence of domain-specific feature on cross-domain object detection.

As shown in Figure 2, our model consists of two parts: a target-domain teacher model and a cross-domain student model. The teacher-student model is trained by mutual learning and adversarial learning. The target domain images are feeding into the teacher model to generate pseudo-labels, which are used to train the student model. The student model is updated with the teacher model by exponential moving average method (EMA). In the student model, we design two branches of feature alignment and feature suppression, respectively. The features are mapped to a high-dimensional space, and the differences between domain-invariant feature in two domain are minimized using a distribution difference measure function. Additionally, the domain-specific feature are constrained to eliminate the influence of domain-specific feature on cross-domain object detection.

The source domain image $x_{i}^{s}$ and the target domain image $x_{i}^{t}$ are as inputs, and the features $F_{S}\left(x_{i}^{s}\right)$ for the source domain and $F_{T}\left(x_{i}^{t}\right)$ for the target domain are obtained by the feature encoder. We design a novel DAB structure. This design allow us to efficiently align domain-invariant feature and suppress domain-specific features from different domains. Basically, our network consists of a target-domain teacher model and a cross-domain student model.

The feature encoder is to ensure that we can construct a feature space of all images, which is used to obtain the feature of the images. The DAB is designed to focuses on domain-invariant feature alignment while suppressing domain-specific feature. Additionally, the detector is used to output the predicted result. In particular, the purpose of the feature alignment branch is to minimize the differences between domain-invariant features using a measure function. The purpose of the feature suppression branch is to eliminate the influence of domain-specific feature.

### 3.2. Optimization Problem in Cross-Domain Student

In cross-domain object detection, we have $N_{s}$ source domains of labeled samples, defined as $D_{S}={\left\{x_{i}^{s},y_{i}^{s}\right\}}_{i=1}^{N_{s}}$, and $N_{t}$ target domains of unlabeled samples, defined as $D_{T}={\left\{x_{i}^{t}\right\}}_{i=1}^{N_{t}}$. The $x_{i}^{s}$ and $x_{i}^{t}$ are denoted as the input samples in the source and target domains, respectively. The $y_{i}^{s}=\left(c_{i}^{s},b_{i}^{s}\right)$ denotes the labels of the corresponding input samples in the source domain, $c_{i}^{s}$ is the category label, and $b_{i}^{s}$ is the bounding box label.

We set the feature of the source domain sample as $F_{S}\left(x_{i}^{s}\right)=\sum_{i=1}^{N_{S}}f_{i}^{s}$, and the feature of the target domain sample as $F_{T}\left(x_{i}^{t}\right)=\sum_{i=1}^{N_{T}}f_{i}^{t}$. The regression representations in the source and target domains are denoted as $P_{S}\left(f_{i}^{s}\right)=\sum_{i=1}^{N_{S}}p_{i}^{s}$ and $P_{T}\left(f_{i}^{t}\right)=\sum_{i=1}^{N_{T}}p_{i}^{t}$, respectively. Our objective is to achieve the same or similar distribution of features in the source and target domain. Therefore, the problem is converted into minimizing the distance of the feature matrix.

Firstly, we map the features in the source and target domains based on the Gaussian distribution. The mean values with different distributions are calculated by finding a continuous function in the sample space, which is used to evaluate the difference in distribution between the source and target domains, as shown in Equations (1) and (2).

(1) $${\hat{F}}_{S}\left(x_{i}^{s}\right)=\frac{1}{N_{S}}\sum_{i=1}^{N_{S}}\sqrt{\frac{2\gamma }{N_{S}!}}{\left(f_{i}^{s}\right)}^{N_{S}}e^{\gamma {\left(f_{i}^{s}\right)}^{2}}$$

(2) $${\hat{F}}_{T}\left(x_{i}^{t}\right)=\frac{1}{N_{T}}\sum_{i=1}^{N_{T}}\sqrt{\frac{2\gamma }{N_{T}!}}{\left(f_{i}^{t}\right)}^{N_{T}}e^{\gamma {\left(f_{i}^{t}\right)}^{2}}$$

where ${\hat{F}}_{S}$ and ${\hat{F}}_{T}$ denote mean of the feature mapping in source and target domains, respectively. $\gamma =-\frac{1}{\delta^{2}}$ denotes the variance of the function.

We use the Frobenius norm to represent the mean of the samples with different distributions, as shown in Equation (3).

(3) $$||{\hat{F}}_{S}-{\hat{F}}_{T}|{|}_{F}=||\frac{1}{N_{S}}\sum_{i=1}^{N_{S}}\sqrt{\frac{2\gamma }{N_{S}!}}{\left(f_{i}^{s}\right)}^{N_{S}}e^{\gamma {\left(f_{i}^{s}\right)}^{2}}-\frac{1}{N_{T}}\sum_{i=1}^{N_{T}}\sqrt{\frac{2\gamma }{N_{T}!}}{\left(f_{i}^{t}\right)}^{N_{T}}e^{\gamma {\left(f_{i}^{t}\right)}^{2}}|{|}_{F}$$

Therefore, the objective function for minimizing the difference in feature distribution can be denoted as Equation (4).

(4) $$\min||{\hat{F}}_{S}-{\hat{F}}_{T}|{|}_{F}$$

**Figure 3:**
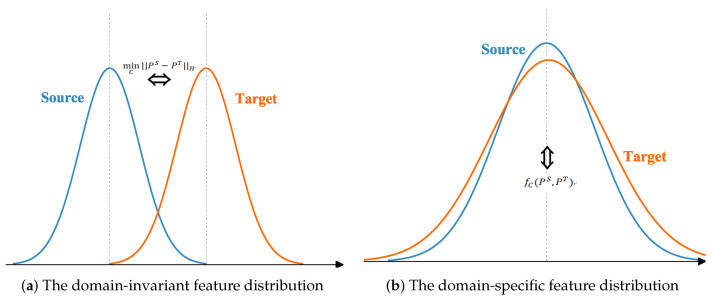
The feature distribution.

As shown in Figure 3a, Equation (4) is used to minimize the domain-invariant feature distribution, which only considers the means of the feature distributions in the source and target domains, but ignores the effect of the variance δ on the feature distributions, as shown in Figure 3b. Therefore, we design a domain adaptive kernel function for aligning the domain-invariant feature distribution and suppressing the domain-specific feature distribution in the source and target domains in parallel.

Therefore, we design $f_{C}\left({\hat{F}}_{S},{\hat{F}}_{T}\right)$ regular terms to suppress the variances of the domain-specific feature distribution, and obtain the optimization problem, as shown in Equation (5).

(5) $$\min||{\hat{F}}_{S}-{\hat{F}}_{T}|{|}_{F}+f_{C}\left({\hat{F}}_{S},{\hat{F}}_{T}\right)$$

We define the domain adaptive kernel function $K(f_{i}^{s},f_{i}^{t})$ as Equation (6).

(6) $$K\left(f_{i}^{s},f_{i}^{t}\right)=e^{-\frac{{\left(f_{i}^{s}+f_{i}^{t}\right)}^{2}}{\delta^{2}}}=e^{\gamma {\left(f_{i}^{s}+f_{i}^{t}\right)}^{2}}=e^{\gamma [{\left(f_{i}^{s}\right)}^{2}+{\left(f_{i}^{t}\right)}^{2}]}e^{\gamma (f_{i}^{s}f_{i}^{t})}$$

Then, we convert Equation (6) to Equation (7) according to Taylor’s formula.

(7) $$K\left(f_{i}^{s},f_{i}^{t}\right)=e^{\gamma [{\left(f_{i}^{s}\right)}^{2}+{\left(f_{i}^{t}\right)}^{2}]}\left[\sum_{i=1}^{n}\frac{2\gamma {\left(f_{i}^{s}f_{i}^{t}\right)}^{n}}{n!}+o{\left(f_{i}^{s}f_{i}^{t}\right)}^{n}\right]$$

When n→∞, we can ignore the residual term to obtain Equation (8).

(8) $$K\left(f_{i}^{s},f_{i}^{t}\right)=\sum_{i=1}^{n}\frac{2\gamma {\left(f_{i}^{s}f_{i}^{t}\right)}^{n}}{n!}e^{\gamma [{\left(f_{i}^{s}\right)}^{2}+{\left(f_{i}^{t}\right)}^{2}]}=\sum_{i=1}^{n}\frac{2\gamma {\left(f_{i}^{s}f_{i}^{t}\right)}^{n}}{n!}e^{\gamma {\left(f_{i}^{s}\right)}^{2}}e^{\gamma {\left(f_{i}^{t}\right)}^{2}}=\sum_{i=1}^{n}\sqrt{\frac{2\gamma }{n!}}{\left(f_{i}^{s}\right)}^{n}e^{\gamma {\left(f_{i}^{s}\right)}^{2}}\sqrt{\frac{2\gamma }{n!}}{\left(f_{i}^{t}\right)}^{n}e^{\gamma {\left(f_{i}^{t}\right)}^{2}}$$

We set,

(9) $$\phi \left({\hat{P}}_{S}\right)=\left[\begin{array}{c} \sqrt{\frac{2\gamma }{n!}}{\left(f_{1}^{s}\right)}^{n}e^{\gamma {\left(f_{1}^{s}\right)}^{2}}\\ \sqrt{\frac{2\gamma }{n!}}{\left(f_{2}^{s}\right)}^{n}e^{\gamma {\left(f_{2}^{s}\right)}^{2}}\\ \vdots \\ \sqrt{\frac{2\gamma }{n!}}{\left(f_{n}^{s}\right)}^{n}e^{\gamma {\left(f_{n}^{s}\right)}^{2}} \end{array}\right],\phi \left({\hat{P}}_{T}\right)=\left[\begin{array}{c} \sqrt{\frac{2\gamma }{n!}}{\left(f_{1}^{t}\right)}^{n}e^{\gamma {\left(f_{1}^{t}\right)}^{2}}\\ \sqrt{\frac{2\gamma }{n!}}{\left(f_{2}^{t}\right)}^{n}e^{\gamma {\left(f_{2}^{t}\right)}^{2}}\\ \vdots \\ \sqrt{\frac{2\gamma }{n!}}{\left(f_{n}^{t}\right)}^{n}e^{\gamma {\left(f_{n}^{t}\right)}^{2}} \end{array}\right]$$

Therefore, we are able to express the domain adaptive kernel function as Equation (10).

(10) $$K\left(f_{i}^{s},f_{i}^{t}\right)=\phi {\left({\hat{P}}_{S}\right)}^{T}\phi \left({\hat{P}}_{T}\right)$$

Then, the optimization problem Equation (5) can be expressed as Equation (11).

(11) $$\min||\frac{1}{N_{S}}\phi \left({\hat{P}}_{S}\right)-\frac{1}{N_{T}}\phi \left({\hat{P}}_{T}\right)|{|}_{F}+||\frac{1}{N_{S}}\phi {\left({\hat{P}}_{S}\right)}^{T}\phi \left({\hat{P}}_{T}\right)-\frac{1}{N_{T}}\phi {\left({\hat{P}}_{T}\right)}^{T}\phi \left({\hat{P}}_{S}\right)|{|}_{2}$$

**Theorem 1.**  *In the kernel mapping operator, we define $||{\hat{F}}_{S}-{\hat{F}}_{T}|{|}_{F}$ and $f_{C}\left({\hat{F}}_{S},{\hat{F}}_{T}\right)$. If $||{\hat{F}}_{S}-{\hat{F}}_{T}|{|}_{F}\to 0$, then we have $f_{C}\left({\hat{F}}_{S},{\hat{F}}_{T}\right)\to 0$.*

**Proof of Theorem 1.**  Since $||{\hat{F}}_{S}-{\hat{F}}_{T}|{|}_{F}\to 0$, we obtain $\phi ({\hat{P}}_{S})$ and $\phi ({\hat{P}}_{T})$, which are similar, and, then, we have $E\left(\phi \left({\hat{P}}_{S}\right)\right)\approx E\left(\phi \left({\hat{P}}_{T}\right)\right)$. By the theorem of expectation, we know that E(Ax)=AE(x), and then $E\left(\phi {\left({\hat{P}}_{S}\right)}^{T}\phi \left({\hat{P}}_{T}\right)\right)\approx E(\phi {\left({\hat{P}}_{T}\right)}^{T}\phi \left({\hat{P}}_{S}\right)$, thus $f_{C}\left({\hat{F}}_{S},{\hat{F}}_{T}\right)\to 0$. □

### 3.3. Feature Alignment Branch in Cross-Domain Student

**Figure 4:**
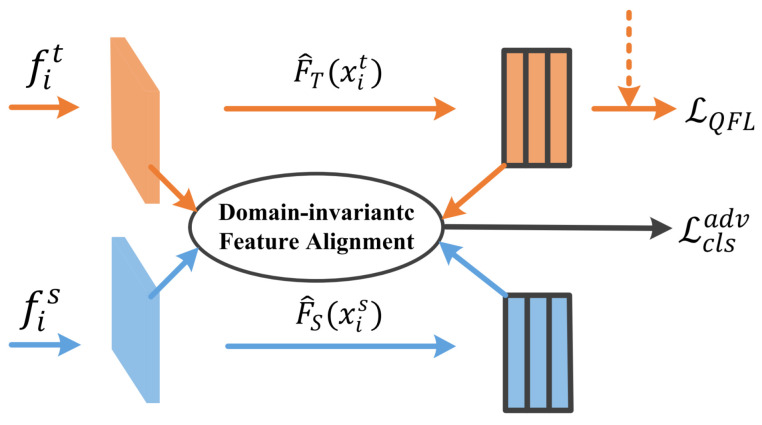
The feature alignment branch.

In the domain adaptive task, it is easy to confuse the object features of different categories, since each image contains multiple objects. Therefore, we propose a feature alignment branch, which aims to minimize the difference of domain-invariant feature between source and target domain, as shown in Figure 4.

The feature encoder obtains the features of the source and target domain, respectively. At first, the quality focal loss is used to calculate the classification loss of the source domain for the source domain samples, which is densely supervised for the whole image.

The objective function is obtained as shown in Equation (12).

(12) $$L_{QFL}\left(P_{S}\left(f_{i}^{s}\right),y_{i}^{gt}\right)=-|y_{i}^{gt}-P_{S}\left(f_{i}^{s}\right){|}^{\beta }\left(1-y_{i}^{gt}\right)\log\left(1-P_{S}\left(f_{i}^{s}\right)\right)+y_{i}^{gt}\log P_{S}\left(f_{i}^{s}\right)$$

where, $y_{i}^{gt}$ denotes the pseudo label from teacher model, the parameter β controls the down-weighting rate smoothly, β=2.

Secondly, we train the feature encoder for obtaining the features $F_{S}\left(x_{i}^{s}\right)$ for the input $x_{i}^{s}$ in the source domain and $F_{T}\left(x_{i}^{t}\right)$ for the input $x_{i}^{t}$ in the target domain, respectively, and we map the feature to ${\hat{F}}_{S}$ and ${\hat{F}}_{T}$, respectively. We use a gradient reversal layer (GRL) in the feature alignment branch, which allows the sign of the gradient to be reversed when the gradient passes through the GRL layer.

We design the feature alignment branch to align the domain-invariant feature in source and target domain. We can derive two distributions, the source anchor distribution $D_{S}^{anc}$ and the target anchor distribution $D_{T}^{anc}$. Therefore, the objective function is obtained as shown in Equation (13).

(13) $$L_{cls}^{adv}=||E_{\left(x_{i}^{s},y_{i}^{gt}\right)∼D_{S}^{anc}}{\hat{F}}_{S}-E_{\left(x_{i}^{t}\right)∼D_{T}^{anc}}{\hat{F}}_{T}|{|}_{F}+f_{C}\left(E_{\left(x_{i}^{s},y_{i}^{gt}\right)∼D_{S}^{anc}}{\hat{F}}_{S}E_{\left(x_{i}^{t}\right)∼D_{T}^{anc}}{\hat{F}}_{T}\right)$$

where we denote each source-domain anchor $x_{i}^{s}\in D_{S}^{anc}$ with a ground truth label $y_{i}^{gt}$.

The Equation (12) is used to guide the feature alignment branch for correct classification in the source domain. Equation (13) is used to encourage the alignment of the mapping ${\hat{F}}_{T}$ on the target domain with the mapping ${\hat{F}}_{S}$ on the source domain, which reduces the difference in the domain-invariant feature distribution. In summary, the objective function of the feature alignment branch is obtained as shown in Equation (14).

(14) $$E_{\left(x_{i}^{s},y_{i}^{gt}\right)∼D_{S}^{anc}}L_{QFL}\left(P_{S}\left(f_{i}^{s}\right),y_{i}^{gt}\right)+\lambda L_{cls}^{adv}$$

where λ is the trade-off parameter.

### 3.4. Feature Suppression Branch in Cross-Domain Student

**Figure 5:**
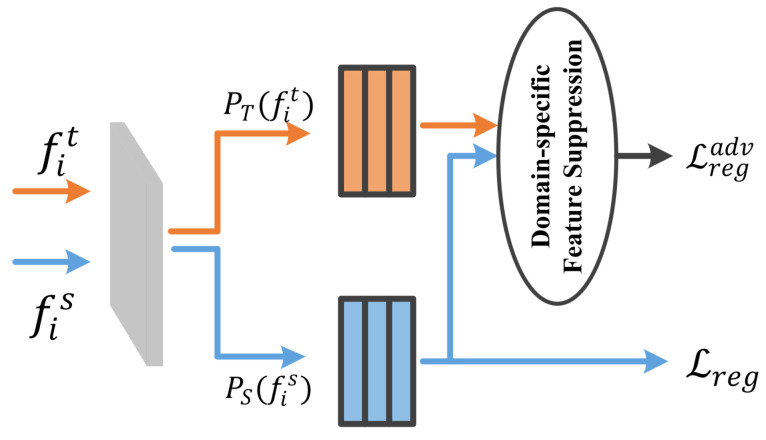
The feature suppression branch.

In the domain adaptive task, we aim to align the domain-invariant feature of two domains while suppressing the domain-specific feature, which gradually converges the feature distributions to the corresponding local optimal values. Therefore, we propose a feature suppression branch for constraining domain-specific feature, as shown in Figure 5. This branch can significantly improve the performance of cross-domain object detection. Especially, the model is well pre-trained on the source domain. The reason is the constraint inhibits the learning of the final task, but the pre-training process performed on the source domain will compensate this limitation. It removes a key obstacle to the cross-domain object detection task.

We have the ground truth category and bounding box labels in each object of the source domain. At first, we calculate the regression loss of the source domain. The objective function is obtained as shown in Equation (15).

(15) $$L_{reg}\left(P_{S}\left(f_{i}^{s}\right),y_{i}^{gt}\right)=1-\frac{|P_{S}\left(f_{i}^{s}\right)\cap y_{i}^{gt}|}{|P_{S}\left(f_{i}^{s}\right)\cup y_{i}^{gt}|}+\frac{\left(\rho^{2}\left(P_{S}\left(f_{i}^{s}\right),y_{i}^{gt}\right)\right)}{c^{2}}+\alpha v$$

where $v=\frac{4}{\pi^{2}}{\left(arctan\frac{w_{i}^{gt}}{h_{i}^{gt}}-arctan\frac{w_{i}^{pre}}{h_{i}^{pre}}\right)}^{2}$ and $\alpha =\frac{v}{(1-IoU)+v}$. $w_{i}^{gt}$ and $h_{i}^{gt}$ denote the width and height of the ground truth bounding box, respectively. $w_{i}^{pre}$ and $h_{i}^{pre}$ denoted the width and height of the prediction bounding box obtained from $P_{T}\left(f_{i}^{t}\right)$, respectively.

The ground truth labels proposed for the target domain are unknown, and we use $P_{T}\left(f_{i}^{t}\right)$ to calculate them. However, it is difficult to obtain a satisfactory detector at the target domain since there is the domain offset. Therefore, we propose a feature suppression branch for domain-specific feature.

In the target domain, we train the feature encoder for obtaining the feature $F_{T}\left(x_{i}^{t}\right)$ of the input $x_{i}^{t}$, and the $P_{T}\left(f_{i}^{t}\right)$ is obtained through the mapping representation ${\hat{F}}_{T}$ of feature $F_{T}\left(x_{i}^{t}\right)$.

With the purpose of feature suppression branch, we measure the differences across domains and suppress the difference of domain-specific feature in source and target domain. The objective function is obtained, as shown in Equation (16).

(16) $$L_{reg}^{adv}=||P_{S}\left(f_{i}^{s}\right)-P_{T}\left(f_{i}^{t}\right)|{|}_{F}+f_{C}\left(P_{S}\left(f_{i}^{s}\right),P_{T}\left(f_{i}^{t}\right)\right)$$

It is noted that the regression loss in the source domain is defined only for the bounding box corresponding to $y_{i}^{gt}$, while $L_{reg}^{adv}$ in the target domain is defined only for the bounding box associated with the predicted category in $P_{T}\left(f_{i}^{t}\right)$. Equation (15) guides the correct prediction in the source domain. Additionally, Equation (16) reduces the difference in the regression representation, which prevents the alignment of the regression representation $P_{T}\left(f_{i}^{t}\right)$ and $P_{S}\left(f_{i}^{s}\right)$ in the target domain. In summary, the objective function of the feature suppression branch is obtained, as shown in Equation (17).

(17) $$L_{reg}\left(P_{S}\left(f_{i}^{s}\right),y_{i}^{gt}\right)+\mu L_{reg}^{adv}$$

where μ is the trade-off parameter.

## 4. Experiment and Analysis

### 4.1. Datasets and Scenarios

#### 4.1.1. Datasets

We perform experiments with popular benchmarks in cross-domain object detection. The details of the datasets are shown in Table 1.

Pascal VOC. It is a dataset collected from the real world and can be used for detection and segmentation. For the detection task, it mainly consists of 20 categories containing 2501 training images, 2510 validation images, and 4952 test images.

Clipart. It is an artistic image created by manual production. Clipart contains 1000 images in a total of 20 categories.

Watercolor. It contains watercolor style images, which consist of images from six categories.

DT Clipart. It uses CycleGAN for style migration, which converts the Pascal VOC dataset to the style of the Clipart dataset. Therefore, the annotation information is identical to the original Pascal VOC dataset.

Cityscapes. It is a semantic segmentation dataset consisting of 2975 training images, 500 validation images, and 1525 test images, each with a size of 1024 × 2048. Each image is annotated at pixel level and can be used for target detection tasks after conversion. The datasets are all urban scenes of different cities under normal weather, and the target objects are mainly pedestrians, vehicles, etc.

Foggy Cityscapes. It is created by adding synthetic fog into the Cityscapes dataset. Therefore, the annotation information is exactly the same as the original Cityscapes dataset.

#### 4.1.2. Scenario

We evaluate our method in two adaptation scenarios.

Dissimilar domains. The purpose is to perform adaptation under dissimilar domains. Firstly, we use the Pascal VOC and Clipart as the source and target domains, respectively. The results are presented on the Clipart val set (Pascal VOC → Clipart). Secondly, we use the Pascal VOC and DT Clipart as the source and target domains, respectively. The results are presented on the DT Clipart test set (Pascal VOC → DT Clipart).

Adverse weather. The purpose is to perform adaptation under different weather conditions. We use the Cityscapes and Foggy Cityscapes as the source and target domains, respectively. The results are presented on the Foggy Cityscapes val set (Cityscapes → Foggy Cityscapes).

### 4.2. Implementation Details

The source code and models were trained and evaluated on the Pytorch toolbox, which is based on the Python 3.6 platform. All experiments were implemented on a NVIDIA RTX 3090Ti GPU. We train the network with a batch size of 16. We use an initial learning rate of 0.125 and a decay rate of 0.1 every 400 K steps. The different scales of detection correspond to different perception fields, and there are a total of 10,647 proposal boxes. We transform the training set in the model by data augmentation, which enriches the training set and enhances the generalization ability. The four images are randomly cropped and scaled, and then randomly arranged and stitched to form a single image. While enriching the dataset, the data of four images are calculated at once during the normalization operation. Therefore, the memory requirement of the model is reduced.

### 4.3. Experimental Results

#### 4.3.1. Adaptation between Dissimilar Domains

Firstly, we have reported the adaptive experiments on dissimilar domains. We use the Pascal VOC dataset as the source domain and the Clipart dataset as the target domain. Source only indicates a model trained with only source domain data, and oracle indicates a model trained with labeled data from the source and target domains. The FA branch only denotes the method with feature alignment branch only, FS branch only denotes the method with feature suppression branch only, and Proposed Method denotes the method with DAB.

As shown in Table 2, we can observe that our method achieves 44.1% mAP with only feature alignment branch (FA branch only), which equals the advanced algorithm UMT [45]. This demonstrates that the feature alignment branch is effective in aligning domain-invariant feature. Additionally, our method achieves 45.9% mAP with only feature suppression branch (FS branch only), which exceeds the advanced algorithm UMT [45] by +1.8%. This illustrates the feature suppression branch can improve detection performance effectively by constraining domain-specific feature. Our method achieves 48.4% mAP with dual adaptive branch (Proposed Method), which outperforms all the other methods, and the detection performance has achieved an improvement of an order of magnitude.

**Figure 6:**
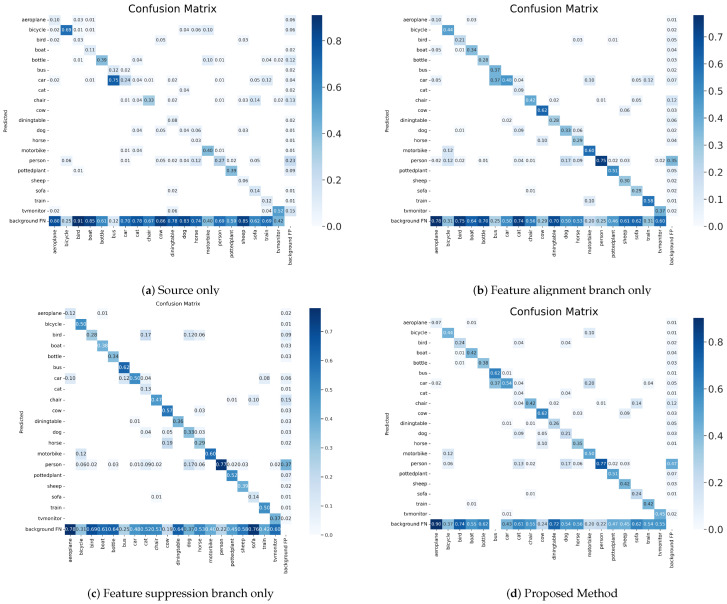
Confusion matrix on Pascal VOC → Clipart transfer.

Although in the experiments mentioned above, our method does not perform best in some categories, such as (‘chair’, ‘cow’, and ‘mbike’). However, we can observe that their differences of average precision is actually not large, and the detection performance of Proposed Method outperforms the method using only feature alignment branch (FA branch only) and only feature suppression branch (FS branch only) in these categories. Additionally, with the confusion matrix in Figure 6, it can be observed that ‘aero’ may be recognized as the ‘bird’ category, and ‘cat’ may be recognized as the ‘dog’ category. We analyze the reasons for the above situation, and it is influenced by the variation of image styles in cross-domain datasets, and it is easy to learn the approximate features in some small sample categories so that the accuracy of detection is reduced.

**Figure 7:**
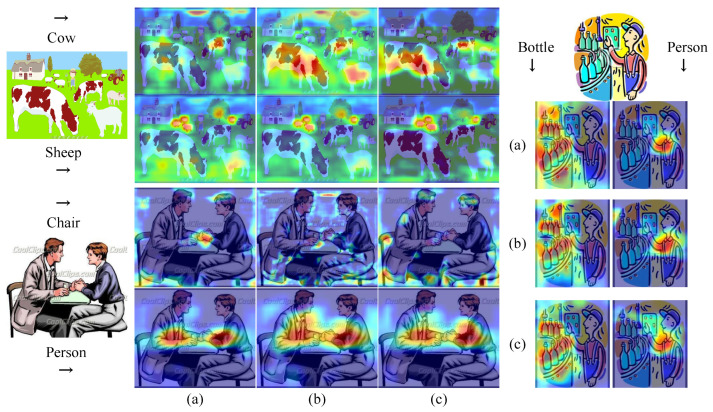
Visualization of the localization ability of multi-label classification. (**a**–**c**) are heat maps of attention for the FA branch only, FS branch only, and Proposed Method, respectively.

Figure 7 shows the heat maps for three exampled images from Clipart, where the main objects related to categories such as “sheep”, “person”, “chair”, “cow”, and “bottle” is localized. Figure 7a–c are heat maps of attention for the FA branch only, FS branch only, and Proposed Method, respectively. It can be observed that the proposed method enables a more accurate alignment of the critical regions and instances. Therefore, it can help the model to activate the main objects of interest more accurately and achieve improved detection performance.

In addition, we use the Pascal VOC dataset as the source domain and the DT Clipart dataset as the target domain. As shown in Table 3, we can observe that our method achieves 52.1% mAP with only the feature alignment branch (FA branch only), our method achieves 53.1% mAP with only the feature suppression branch (FS branch only), and our method achieves 54.7% mAP with dual adaptive branch (Proposed Method), which outperforms all the other methods and the detection performance has achieved the order of magnitude improvement.

We will further evaluate the generalization ability of our model on unknown domains. We trained the model on the source domain dataset with labels (PASCAL VOC) and another artistic dataset without labels (Watercolor). Then, we inferred the model on a target dataset (Clipart), which is unknown during training. We only trained the overlapping classes (six classes) between Clipart1k and Watercolor, and the results are given in Table 4.

Compared with AT [51] and MT [50], our model achieved the best performance in several categories. Despite this, in some categories (such as bicycle, dog, and person), our method is not achieving the optimal performance, but we can observe that their differences of average precision is actually not large. It demonstrates that our model can promote to unknown domains.

#### 4.3.2. Adaptation between Adverse Weather

We have reported the experimental results of domain adaptive object detection under adverse weather condition. Source only indicates a model trained with only source domain data, and oracle indicates a model trained with labeled data from the source and target domains.The FA branch only denotes the method with feature alignment branch only, FS branch only denotes the method with feature suppression branch only, and Proposed Method denotes the method with DAB.

Table 5 shows the experimental results on Cityscapes → Foggy Cityscapes transfer. We can observe that our method achieves 43.6% mAP with feature alignment branch only (FA branch only), our method achieves 43.5% mAP with feature suppression branch only (FS branch only), and our method achieves 47.4% mAP with DAB (Proposed Method). It is worth noting that the mAP is approximated in the FA branch only method and the FS branch only method. However, in the categories of pedestrians and riders, the detection performance of the FS branch only method is better. The state-of-the-art method TDD [52] achieves 43.1% mAP, while our method achieves +4.3% gains. It shows that our method has the stable ability to solve the domain adaptive problem in adverse weather.

**Figure 8:**
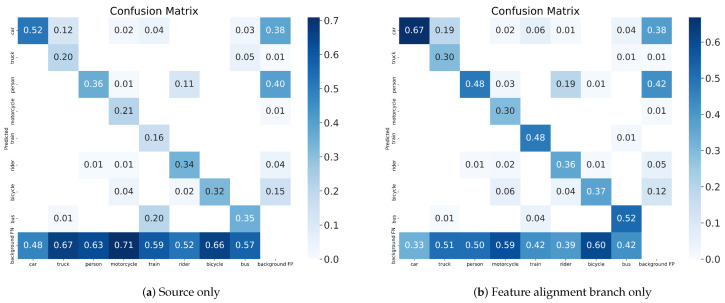

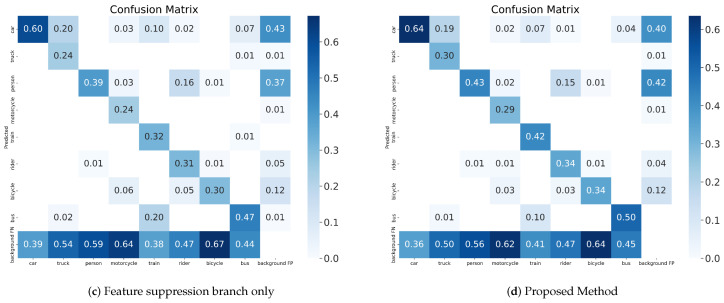
Confusion Matrix on Cityscapes → Foggy Cityscapes transfer.

The confusion matrix for the source only method and our method are shown in Figure 8. We can clearly observe the improvement in detection quality. The results show that the proposed method significantly improves the performance, especially for the accuracy of localization.

### 4.4. Ablation Study

In this study, we investigate the performance of various strategies for aligning feature representations. We have used Pascal VOC → Clipart to conduct the study. For a fair comparison, all experiments have been performed under the same settings.

**Trade-off parameter.** Firstly, we investigate the effect of different trade-off parameters for the performance in domain adaptive object detection. We present the results of the ablation experiments for the trade-off parameter in Table 6.

We can observe that the trade-off parameter of EXP.2 achieves optimal performance. Both $AP_{0.5}$ and $AP_{0.5:0.95}$ outperformed the results of EXP.1 and EXP.3, with gains of +4.7% and +0.9%, and +2.7%, and +0.2%, respectively.

**Scales.** There is a potential scale shift between the source and target domain datasets. To investigate the effect of image scale on our method, we have changed the size of the image in the target domain, and the scale in the source domain is fixed at 640 pixels. We have plotted the detection performance at different image scales by changing the scales of the target domain images. The FA branch only denotes the method with feature alignment branch only, FS branch only denotes the method with feature suppression branch only, w/o DAB denotes the method without the DAB, and Proposed Method denotes the method with DAB.

**Figure 9:**
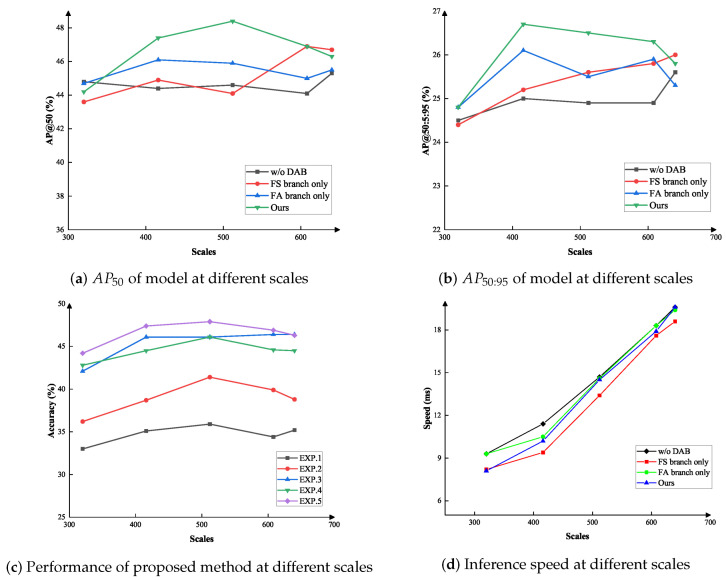
The ablation study for the scales of different target domain inputs. The results are from Pascal VOC → Clipart. The scale of image is fixed to be 640 pixels in the source domain, and we resize the different scales of image in the target domain, as shown in the X-axis.

As shown in Figure 9a,b, we observe that changing the scales under the same experimental conditions, the model with the DAB achieves better results at most scales. In Figure 9c, we can observe the detection performance of our model with the DAB at each scale. EXP.1-EXP.5 indicate the experiments were performed at various model depth conditions. In Figure 9d, we can observe the inference speed at different scales.

Comparing the two branch, we observe that the feature alignment branch is more robust to scale variation than the feature suppression branch. The reason is that scale variation is a global shift that affects all objects and backgrounds. While, in our method, the global domain shift is mainly solved by feature alignment branch to domain-invariant feature alignment, and the feature suppression branch is used to constraint domain-specific feature. When a significant global domain shift is present, the localization error is increased, and therefore the accuracy of the feature suppression branch is affected by the error in the domain-specific feature. Nevertheless, DAB consistently provide the optimal results at all scales.

**Conv kernel.** The depth of the model might affect the performance of feature extraction. To investigate the effect of different model depths for our method, we conduct experiments with different convolution kernel conditions. The reported results are set with Pascal VOC → Clipart. We conducted experiments at the same image scale as shown in Table 7.

**Figure 10:**
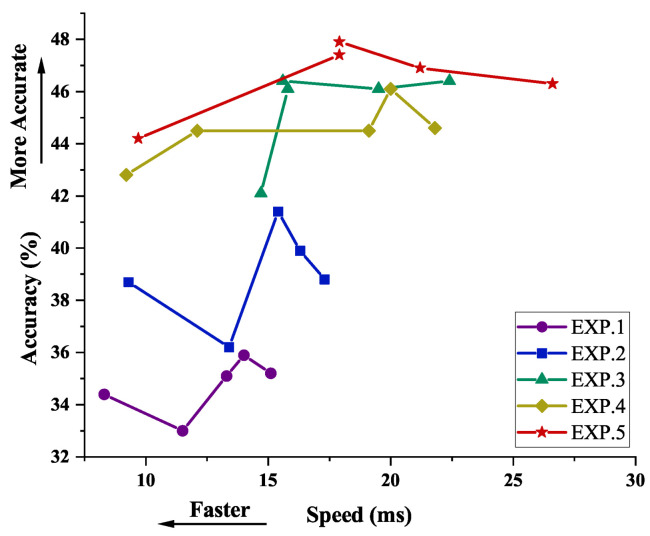
The ablation study of different network depths. The results are from Pascal VOC → Clipart. The image scale has been fixed at 640 pixels, and we evaluated the accuracy and speed of different depth networks.

We can observe that the EXP.5 obtained the optimal performance, the $AP_{0.5}$ and $AP_{0.5:0.95}$ achieved 46.2% and 25.9% mAP, respectively. We also report the results of different convolution kernels in Figure 10, which evaluates the accuracy and speed of different convolution kernel models. It can be observed that, for trade-off between accuracy and speed, EXP.5 also achieves optimal performance.

**Branch structure.** To verify the effectiveness of our dual adaptive branch structure, we have performed a set of ablation studies. The reported results are set with Pascal VOC → Clipart. The results of the different experiments are shown in Table 8.

We use Proposed Method as the baseline. It can be observed that Proposed Method outperforms FA branch only in $AP_{0.5}$ and $AP_{0.5:0.95}$, achieving the gains of +2.5% and +0.9%, respectively. The Proposed Method outperforms the FS branch only in $AP_{0.5}$ and $AP_{0.5:0.95}$, achieving gains of +4.3% and +1.1%, respectively. The Proposed Method outperforms all the above experiments in $AP_{0.5}$ and $AP_{0.5:0.95}$, achieving 48.4% and 26.5% mAP. It demonstrates that the model performance is gradually improved with DAB involved in the training, which illustrates the utility of each branch. The experiment of DAB is superior to all single branch methods, which indicates that our method preserves useful source domain knowledge effectively and explores target domain information in parallel.

### 4.5. Error Analysis

To create a meaningful distribution of errors and identify the components of the mAP, we separated all false positives and false negatives of model with four types. We will represent the overlap between the maximum IoU of a false positive and the ground truth of a given category as IoUmax. The foreground IoU threshold is denoted as $t_{f}$, the background threshold is denoted as $t_{b}$, and the above thresholds are set at 0.5 and 0.1, respectively [57]. $IoU_{max}\geq t_{f}$ is denoted as classification error (Cls), which indicates localized correctly but classified incorrectly. $t_{b}\leq IoU_{max}\leq t_{f}$ is denoted as localization error (Loc), which indicates classified correctly but localized incorrectly. $IoU_{max}\leq t_{b}$ is denoted as background error (Bkg), which indicates detected background as foreground. Additionally, missed GT error (Miss) indicates undetected ground truth.

**Figure 11:**
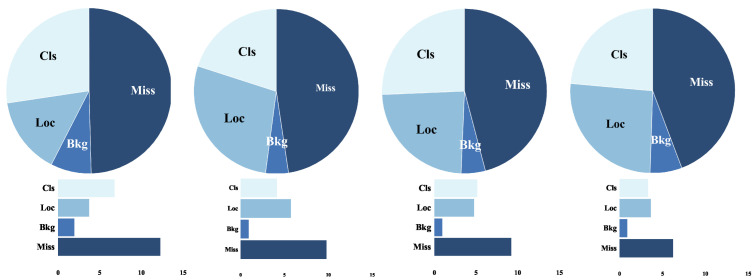
Error analysis. Results are reported using the Pascal VOC → Clipart setup. Four types: Cls (classification error), Loc (localization error), Bkg (background error), and Miss (missed GT error). From left to right, it is source only, feature alignment branch only, feature suppression branch only, and DAB, respectively.

The error ratios for each model on Pascal VOC → Clipart are shown in Figure 11. It can be observed that the main errors in the target domain appear from: Miss (undetected ground truth), Cls (incorrect classification), and Loc (incorrect localization). As shown in column 2 of Figure 11, it is observed that the error ratio of Cls is effectively reduced after feature alignment branch, but the error ratio of Loc is increased. This also illustrates the necessity for the feature suppression branch is performed. As shown in column 3 of Figure 11, it can be observed that the error ratio of Loc effectively decreases through the constraint domain-specific feature. In summary, it is illustrated that DAB is reasonable.

### 4.6. Visualisation Results

**Figure 12:**
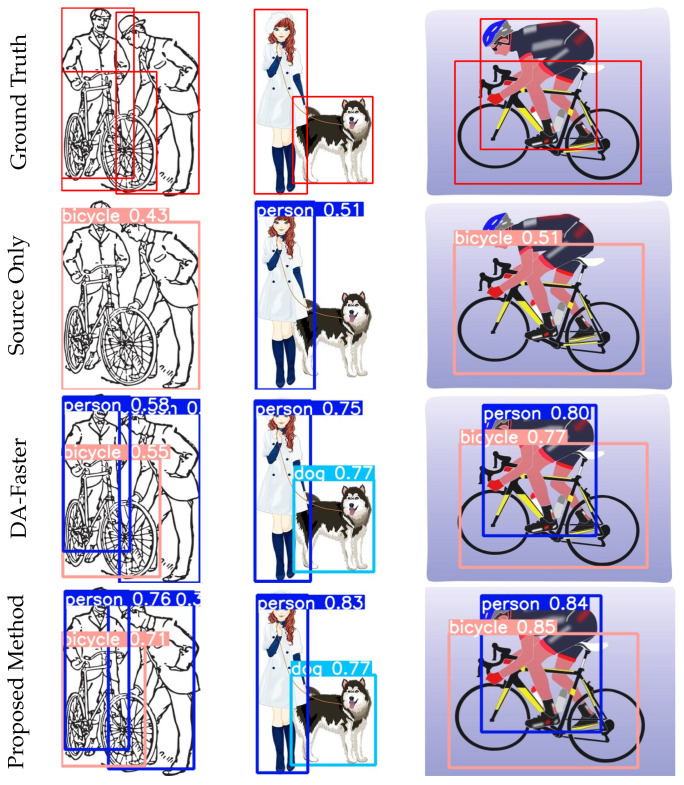
Qualitative results (Pascal VOC → Clipart). The Pascal VOC dataset is used as source domain, and the Clipart dataset is used as target domain. From left to right are the results from ground truth, source only, DA-Faster, and proposed method.

Figure 12 shows the qualitative results in Pascal VOC → Clipart cross-domain detection. From top to bottom, the visualization results of ground truth, source only, DA-faster [5], and proposed method are shown, respectively. We can observe that there are many missing and incorrect results in the cource only. Compared with DA-faster [5], our method has more significant improvement in localization accuracy, which indicates that the problem of errors and false positives has been improved. We can clearly observe the improvement of the detection quality. The results demonstrate a significant improvement of performance with our method.

**Figure 13:**
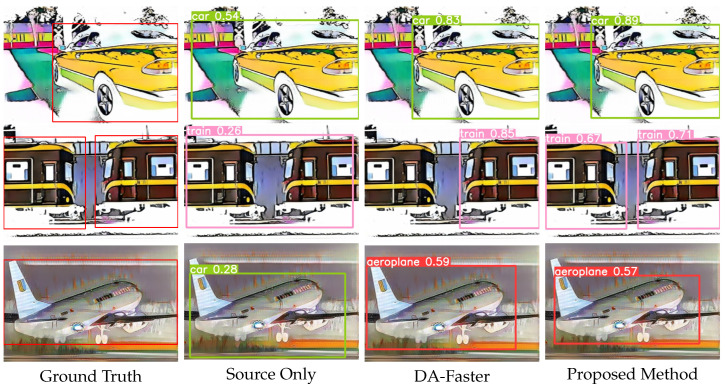
Qualitative results (Pascal VOC→DT Clipart). The Pascal VOC dataset is used as source domain, and the DT Clipart dataset is used as target domain. From left to right are the results from ground truth, source only, DA-Faster, and proposed method.

**Figure 14:**
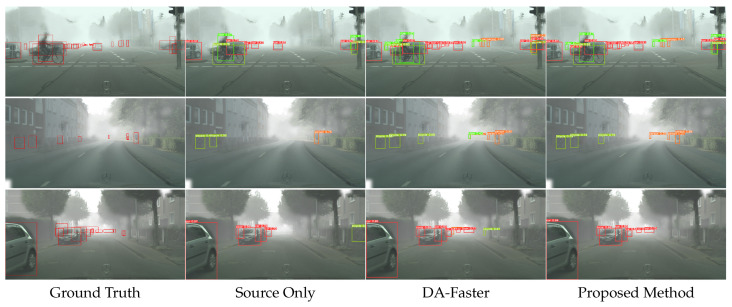
Qualitative results (Cityscapes→Foggy Cityscapes). The Cityscapes dataset is used as source domain, and the Foggy Cityscapes dataset is used as target domain. From left to right are the results from ground truth, source only, DA-Faster, and proposed method.

Figure 13 shows the qualitative results in Pascal VOC → DT Clipart cross-domain detection. Figure 14 shows the qualitative results in Cityscapes → Foggy Cityscapes cross-domain detection. From left to right, the visualization results of ground truth, source only, DA-faster and proposed method are shown, respectively. We can observe that the detection results of source only have incorrect detection and mis-location. In the DA-Faster [5] detection results, there are several omissions. Additionally, our method significantly improves the appearance of the above situations. Notably, our method shows a competitive performance with the oracle model. This demonstrates that our model can perceive the knowledge of the target domain while retaining the useful information of the source domain.

## 5. Conclusions

In this work, we address the domain shift in high-level semantic features by proposing a novel DAB structure. The purpose of domain adaptation is to focus on domain-invariant features and to eliminate the interference of domain-specific features. Therefore, we propose feature alignment and feature suppression branches, respectively. The effect of feature shift is eliminated by this strategy, which reduces the probability of false positives and errors in detection. Specifically, we exploit a distribution difference metric function to improve prediction consistency. It allows the model to focus on the object-relevant features aligned in the high-level semantic space. Experimental results on the common benchmarks indicated that our model achieves a comparable performance with advanced methods, achieving 48.4% mAP and 47.4% mAP, outperforming the next best methods by +1.8% and +4.3%, respectively. The experimental results also indicated that our detector is highly robust in different scales, which is very effective and advantageous in cross-domain object detection.

## Figures and Tables

**Table 1:** The details of samples in the datasets.

Datasets	Train	Val	Test	Total
Pascal VOC [42]	2501	2510	4952	9963
Clipart [43]	600	200	200	1000
Watercolor [43]	1000	500	500	2000
DT Clipart [43]	2501	2510	4952	9963
Cityscapes [44]	2975	500	1525	5000
Foggy Cityscapes [44]	2975	500	1525	5000

**Table 2:** The mean Average Precision (mAP) of different models on Clipart val set for Pascal VOC → Clipart transfer. The best results are in bold.

Methods	Aero	Bike	Bird	Boat	Bottle	Bus	Car	Cat	Chair	Cow	Table	Dog	Horse	Mbike	Person	Plant	Sheep	Sofa	Train	TV	mAP
DA-Faster [5]	15.0	34.6	12.4	11.9	19.8	21.1	23.2	3.1	22.1	26.3	10.6	10.0	19.6	39.4	34.6	29.3	1.0	17.1	19.7	24.8	19.8
SWDA [6]	26.2	48.5	32.6	33.7	38.5	54.3	37.1	18.6	34.8	58.3	17.0	12.5	33.8	65.5	61.6	52.0	9.3	24.9	**54.1**	49.1	38.1
SCL [46]	**44.7**	50.0	33.6	27.4	42.2	55.6	38.3	**19.2**	37.9	69.0	30.1	26.3	34.4	67.3	61.0	47.9	21.4	26.3	50.1	47.3	41.5
HTCN [47]	33.6	58.9	34.0	23.4	**45.6**	57.0	39.8	12.0	39.7	51.3	21.1	20.1	39.1	72.8	63.0	43.1	19.3	30.1	50.2	51.8	40.3
ATF [40]	41.9	67.0	27.4	36.4	41.0	48.5	42.0	13.1	39.2	**75.1**	33.4	7.9	41.2	56.2	61.4	50.6	42.0	25.0	53.1	39.1	42.1
UMT [45]	39.6	59.1	32.4	35.0	45.1	61.9	48.4	7.5	**46.0**	67.6	21.4	29.5	**48.2**	**75.9**	70.5	56.7	25.9	28.9	39.4	43.6	44.1
Source only	5.4	50.2	7.9	15.9	40.6	16.2	22.1	0.3	36.8	1.7	25.6	8.3	10.0	36.7	34.8	46.4	11.0	24.0	10.1	31.3	21.8
FA branch only	13.7	67.4	33.3	38.6	33.1	63.9	52.3	15.5	40.3	54.4	34.8	28.3	42.1	53.8	76.2	56.3	41.9	25.1	52.6	**56.8**	44.1
FS branch only	27.7	60.6	**38.6**	**48.3**	42.8	65.1	**59.3**	16.6	41.4	52.9	40.5	**30.4**	36.8	59.3	75.7	56.8	**48.0**	22.5	49.5	44.9	45.9
Proposed Method	25.2	**75.7**	31.8	42.3	32.5	**70.8**	57.2	18.3	42.2	73.7	**42.5**	25.7	41.1	65.9	**77.4**	**58.0**	47.9	**33.7**	52.5	53.4	**48.4**
oracle	55.2	78.3	51.1	58.1	60.7	58.4	61.5	27.3	60.9	71.7	60.5	40.7	56.9	82.5	82.8	65.9	49.2	46.1	59.7	58.1	59.3

**Table 3:** The mean Average Precision (mAP) of different models on DT Clipart test set for Pascal VOC → DT Clipart transfer. The best results are in bold.

Methods	Aero	Bike	Bird	Boat	Bottle	Bus	Car	Cat	Chair	Cow	Table	Dog	Horse	Mbike	Person	Plant	Sheep	Sofa	Train	TV	mAP
ADDA [48]	20.1	50.2	20.5	23.6	11.4	40.5	34.9	2.3	39.7	22.3	27.1	10.4	31.7	53.6	46.6	32.1	18.0	21.1	23.6	18.3	27.4
CDWS [49]	23.3	60.1	24.9	**41.5**	26.4	53.0	44.0	4.1	**45.3**	51.5	39.5	11.6	40.4	62.2	61.1	37.1	20.9	39.6	38.4	36.0	38.0
Source only	4.4	44.9	7.8	15.6	**33.5**	27.1	22.1	0.4	37.9	9.2	23.5	10.4	13.6	40.8	40.3	**43.0**	17.0	14.7	23.6	40.6	23.5
FA branch only	64.6	77.2	40.7	29.7	26.0	61.6	77.8	58.5	33.5	48.9	53.0	44.8	70.5	**77.7**	69.8	27.7	37.8	38.6	62.2	41.9	52.1
FS branch only	62.0	75.8	37.3	26.5	31.3	68.2	**80.2**	60.2	37.3	**53.2**	52.3	41.8	63.7	76.3	70.5	33.1	38.0	40.4	67.2	46.4	53.1
Proposed Method	**67.1**	**78.5**	**39.2**	30.6	26.3	**70.5**	76.9	**63.8**	37.7	52.3	**57.8**	**45.1**	**68.3**	74.4	**71.0**	30.5	**45.1**	**43.4**	**67.3**	**47.3**	**54.7**

**Table 4:** The domain generalization ability on unknown domains. The best results are in bold.

Methods	Pascal VOC & Watercolor → Clipart					
	Bicycle	Bird	Car	Cat	Dog	Person
MT [50]	64.8	23.4	34.6	3.1	22.0	61.4
AT [51]	**78.6**	30.1	40.3	10.9	**32.6**	**72.8**
Proposed Method	60.4	**30.6**	**52.9**	**14.4**	22.4	66.5

**Table 5:** The mean Average Precision (mAP) of different models on Foggy Cityscapes val set for Cityscapes → Foggy Cityscapes transfer. The best results are in bold.

Methods	Person	Rider	Car	Truck	Bus	Train	Motor	Bike	mAP
DA-Faster [5]	31.9	41.6	46.4	20.1	32.0	17.5	23.1	34.6	30.9
SCDA [37]	33.5	38.0	48.5	26.5	39.0	23.3	28.0	33.6	33.8
SWDA [6]	29.9	42.3	43.5	24.5	36.2	32.6	30.0	35.3	34.3
MTOR [39]	30.6	41.4	44.0	21.9	38.6	40.6	28.3	35.6	36.0
iFan [53]	32.6	40.0	48.5	27.9	45.5	31.7	22.8	33.0	35.3
HTCN [47]	33.2	47.5	47.9	31.6	47.4	40.9	32.3	37.1	39.8
GPA [24]	32.9	46.7	54.1	24.7	45.7	41.1	32.4	38.7	39.5
SFA [54]	46.5	**48.6**	62.6	25.1	46.2	29.4	28.3	**44.0**	41.3
UMT [45]	33.0	46.7	48.6	34.1	56.5	46.8	30.4	37.3	41.7
AFAN [55]	42.5	44.6	57.0	26.4	48.0	28.3	33.2	37.1	39.6
DIR [56]	36.9	45.8	49.4	28.2	44.6	34.9	35.1	38.9	39.2
TDD [52]	39.6	47.5	55.7	33.8	47.6	42.1	**37.0**	41.4	43.1
Source only	27.6	31.4	48.9	21.2	33.8	16.9	19.9	23.1	27.9
FA branch only	40.4	41.7	64.8	30.6	55.3	**56.7**	25.9	33.5	43.6
FS branch only	41.2	42.1	64.2	32.0	54.4	49.9	29.8	34.1	43.5
Proposed Method	**46.1**	46.5	**68.9**	**35.6**	**57.1**	50.8	35.2	38.7	**47.4**
oracle	51.2	49.2	71.9	40.1	57.7	56.3	40.1	42.3	51.1

**Table 6:** The ablation study of the trade-off parameter. The best results are in bold.

EXP.	λ	μ	Scale	${\mathrm{AP}}_{0.5}$	${\mathrm{AP}}_{0.5:0.95}$	Speed/ms
1	0.99	0.99	512	43.2	23.8	14.9
2	0.099	0.099	512	**48.4**	**26.5**	23.7
3	0.0099	0.0099	512	47.0	26.3	19.2

**Table 7:** The ablation study of Conv kernel.

EXP.	Conv Kernel	Scale	Params/M	${\mathrm{AP}}_{0.5}$	${\mathrm{AP}}_{0.5:0.95}$	Speed/ms	FLOPs/B
1	[16,32,64,128,256]	640	1.79	35.2	17.4	15.1	4.2
2	[32,64,128,256,512]	640	7.1	38.8	19.5	17.3	15.9
3	[48,96,192,384,768]	640	20.9	46.4	22.4	22.4	48.1
4	[64,128,256,512,1024]	640	46.2	44.4	23.6	19.1	108.6
5	[80,160,320,640,1280]	640	86.3	46.2	25.9	37.4	204.2

**Table 8:** The ablation study of the DAB structure.The best results are in bold.

Method	Scale	${\mathrm{AP}}_{0.5}$	${\mathrm{AP}}_{0.5:0.95}$	Speed/ms
FA branch only	512	45.9	25.6	14.2
FS branch only	512	44.1	25.4	14.7
Proposed Method	512	**48.4**	**26.5**	23.7

## Data Availability

Not applicable.
